# Intramedullary Epidermoid Cyst Recurrence Diagnosed With Computed Tomography Myelography: A Case Report

**DOI:** 10.1155/crnm/3763090

**Published:** 2025-12-25

**Authors:** Leonardo Favi Bocca, Alexandre Israel Kochi Silva, Thiago Bortholin, Thiago Pereira Rodrigues, Igor Almeida de Oliveira, Fabio Veiga de Castro Sparapani, Sergio Cavalheiro, João Norberto Stavale, Franz Jooji Onishi

**Affiliations:** ^1^ Department of Neurology and Neurosurgery, Universidade Federal de São Paulo, São Paulo, Brazil, unifesp.br; ^2^ Division of Neurosurgery, Pirajussara General Hospital, Taboão da Serra, Brazil; ^3^ Department of Pathology, Universidade Federal de São Paulo, São Paulo, Brazil, unifesp.br

**Keywords:** artifacts, computed tomography, epidermoid cyst, myelography

## Abstract

The introduction of computed tomography (CT) myelography for spinal diseases has allowed the diagnosis of several intradural and extradural etiologies. With the advent of water‐soluble, nonionic contrast agents, the safety and availability of this technique have expanded. Although magnetic resonance imaging (MRI) has faded the indications for CT myelography, some specific conditions and settings still benefit from the functional flow of contrast in the subarachnoid space and/or the patient’s particular limitations in performing MRI (especially in the presence of intense metallic artifacts). We present the case of a 23‐year‐old male patient who underwent long‐term follow‐up for an intramedullary epidermoid cyst. At the time of diagnosis, the patient complained of right lower limb tremor and pain, which progressed to leg weakness, with no congenital abnormality. His first surgery resulted in a mass resection without spinal fixation. Three years later, thoracic canal stenosis and kyphosis were diagnosed, leading to a second surgery, consisting of laminectomy and cervicothoracic fixation. At 8 years of age, worsening weakness and sphincter issues prompted further evaluations. CT myelography revealed upper thoracic cord enlargement. An intramedullary epidermoid cyst was diagnosed, and the patient underwent a new gross total resection. CT myelography is not obsolete. Patients requiring spine imaging for oncologic control and/or new or worsening neurological symptoms may benefit from CT myelography when standard spine MRI cannot be performed. Epidermoid cysts require long‐term postoperative follow‐up, as recurrence may occur years after surgical resection owing to their indolent, benign behavior.

## 1. Introduction

The introduction of the computed tomography (CT) myelography technique in 1976 by Di Chiro and Schellinger revolutionized the diagnosis of a myriad of spinal diseases [1]. This technique enables detailed visualization of intradural‐extramedullary cysts, meningeal diseases, cerebrospinal fluid (CSF) leaks, and nerve root avulsions, particularly pathologies affecting the thecal sac and its contents [1]. This method is similar to the conventional myelography method [2]. CT image acquisition is performed after intrathecal radiopaque contrast injection, delineating the spinal cord and nerve roots, and space‐occupying conditions. In the 1970s and the 1980s, substitution of iodized oil contrast agents with water‐soluble nonionic contrast agents made the method easier and safer to perform [3].

In recent years, the role of CT myelography in diagnosis has decreased, mainly because of the wide availability of magnetic resonance imaging (MRI) [1, 3], making CT myelography almost obsolete. Although MRI is the modality of choice for the evaluation of nervous tissue and other soft tissue components, the functional factor of iodine contrast flow from the injection point to all subarachnoid spaces provides a particular value in assessing subarachnoid space patency and communication, especially in cases of arachnoid adhesions or cysts.

The most accepted categorization of spinal canal tumors has divided the neoplasms anatomically into three groups: intramedullary, intradural‐extramedullary, and extradural [4]. The overall global incidence of primary spinal tumors, both benign and malignant, is 0.098 cases per 100,000 person‐years, accounting for approximately 4%–8% of all central nervous system tumors [5]. Among benign spinal tumors, epidermoid cysts are rare, with only 139 reported cases [6]. Recurrent epidermoid cysts are an even rarer outcome, with a similar clinical presentation of slow‐progressing neurological impairment and back pain [6, 7].

This report presents a case of a recurrent epidermoid cyst diagnosed through CT myelography, highlighting the continued utility of this imaging modality in specific clinical scenarios.

## 2. Case Presentation

This case report was conducted by institutional ethical guidelines and received approval from the Institutional Review Board (CAAE: 77396324.7.0000.5505). The patient provided written informed consent for the publication of this report.

A 23‐year‐old male patient was admitted to our institution’s Neurosurgery Department and was followed up for a long time after the diagnosis of a thoracic intramedullary epidermoid cyst. The initial presentation included spontaneous right lower limb tremors and pain. The patient had no significant previous medical history, including no reported surgery or trauma in the back region, and skin inspection was unremarkable for any spina bifida occulta–related stigma. After 2 years, the symptoms progressed to weakness in both legs, which led to the patient being referred to our institution. Spinal MRI evaluation was performed (Figure 1), which revealed an intramedullary spinal cord tumor in the thoracic level. The first surgical procedure involved gross total mass resection and return and fixation of the posterior spinal elements to the operated level (osteoplastic laminotomy). To mitigate the risks associated with iatrogenic segmental kyphosis, the procedural plan emphasizes maintaining the integrity of the posterior ligamentous complex as much as possible. Ideally, this involves preserving the connection of the lamina to the most cranial or caudal vertebra and avoiding injury to the facet joints. When feasible, a unilateral laminectomy may be employed, which allows for the preservation of the contralateral paraspinal muscles and ligaments, which was not possible in this presented case due to no clear lateralization of intramedullary mass. Torso and lower limb paresthesia persisted, with weakness progressively improving to normality.

Figure 1Preoperative T2‐weighted spine MRI. (a, b) Intramedullary spinal cord tumor (epidermoid cyst) characterized by a hyperintense, heterogenic mass in the thoracic spinal cord (arrow, (a)), narrowing and peripherally spreading spinal cord (b). (c) Late postoperative T2‐weighted MRI showing segmental stenosis and chronic spinal signal changes (arrow). (d) Postoperative spine CT, arrows pointing to the returned laminotomy segment.

**Figure:**
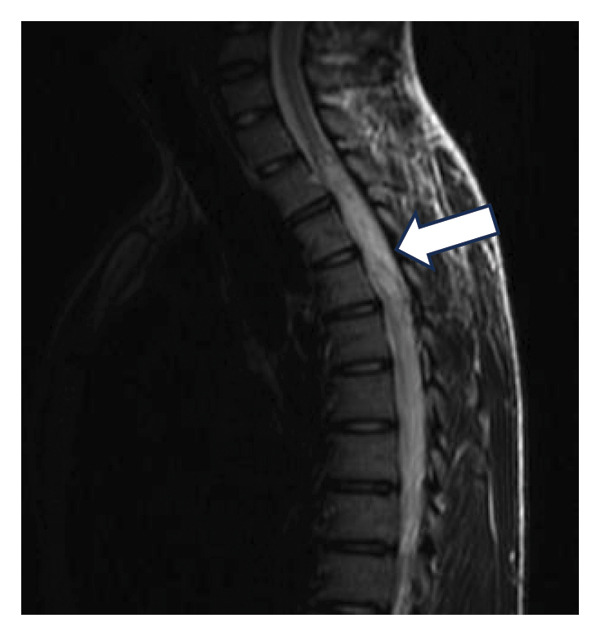
(a)

**Figure:**
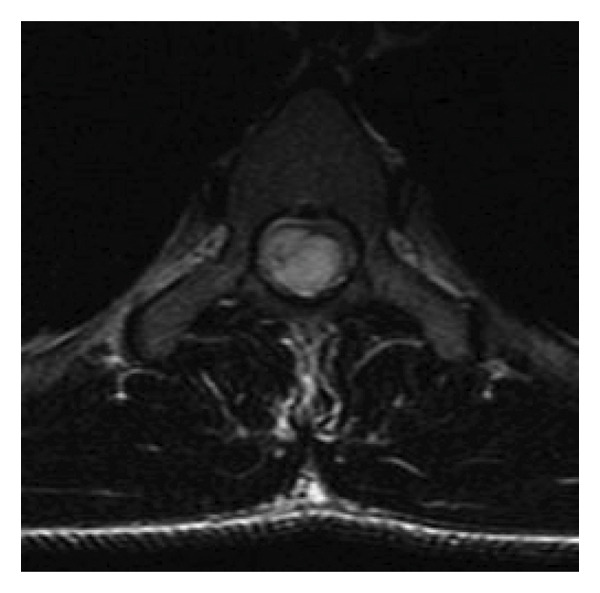
(b)

**Figure:**
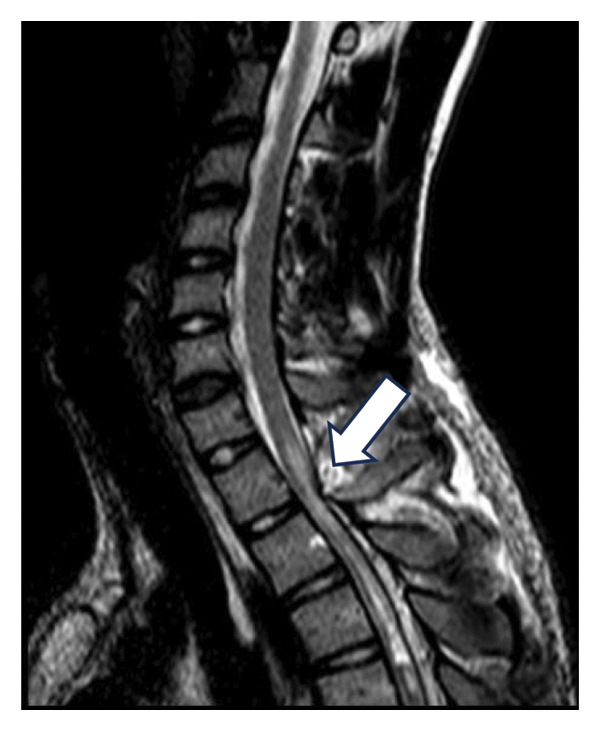
(c)

**Figure:**
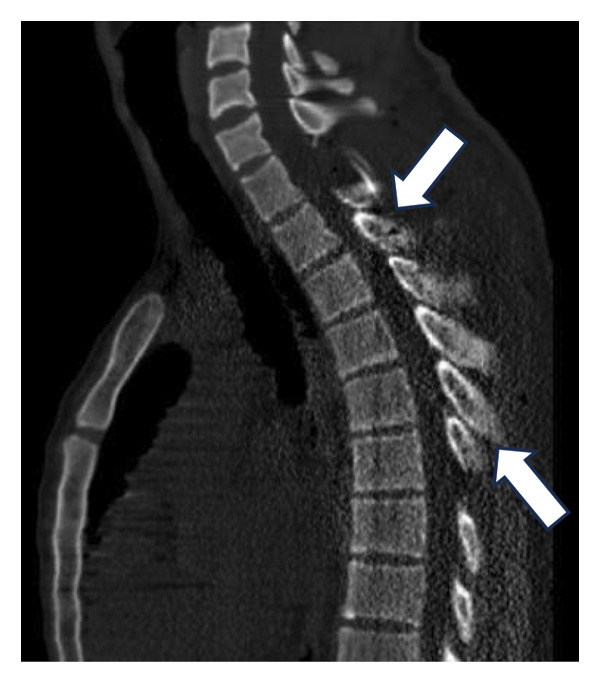
(d)

During follow‐up, new‐onset lower limb paresis at 3 years of follow‐up required image re‐evaluation, and a diagnosis of canal stenosis associated with segmental kyphosis in the upper thoracic segment (Figure 1(c)) was made. The patient underwent a second surgical procedure, which involved a broad laminectomy and cervicothoracic fixation (Figure 2). Posterior fixation was performed because of instability in the operated segment, as evidenced by thoracic kyphosis.

Figure 2Posteroanterior (a) and lateral (b) projections of the cervicothoracic spine. Final aspects of laminectomy and cervicothoracic fixation. Note the density of the metal around the spinal canal and the thoracic kyphosis.

**Figure:**
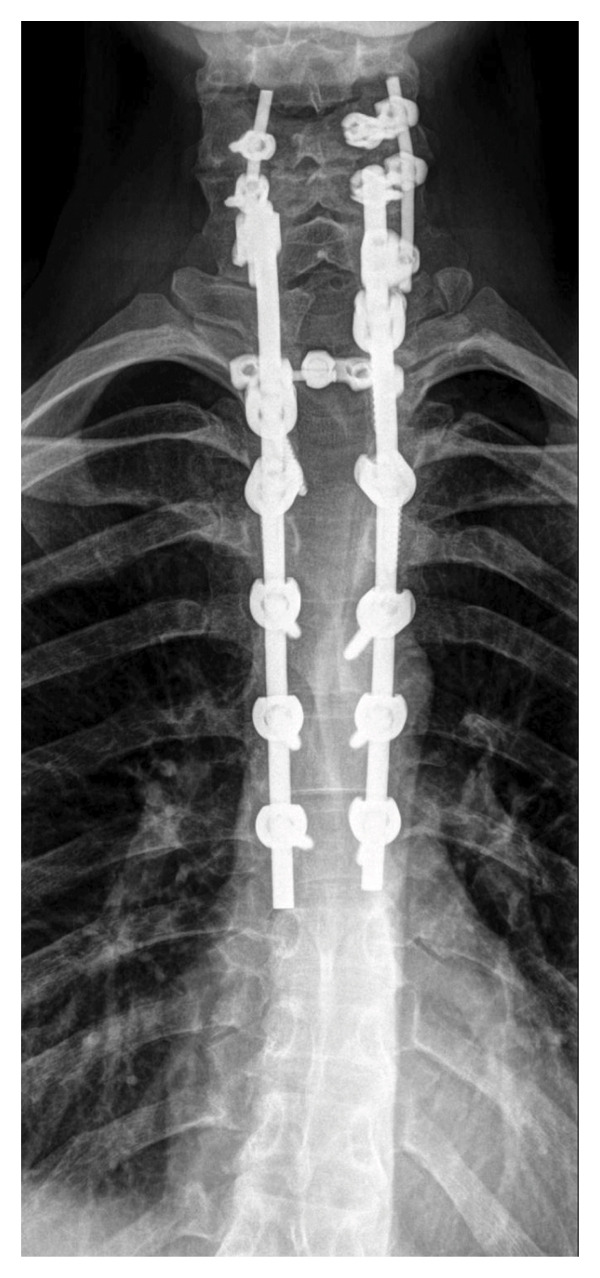
(a)

**Figure:**
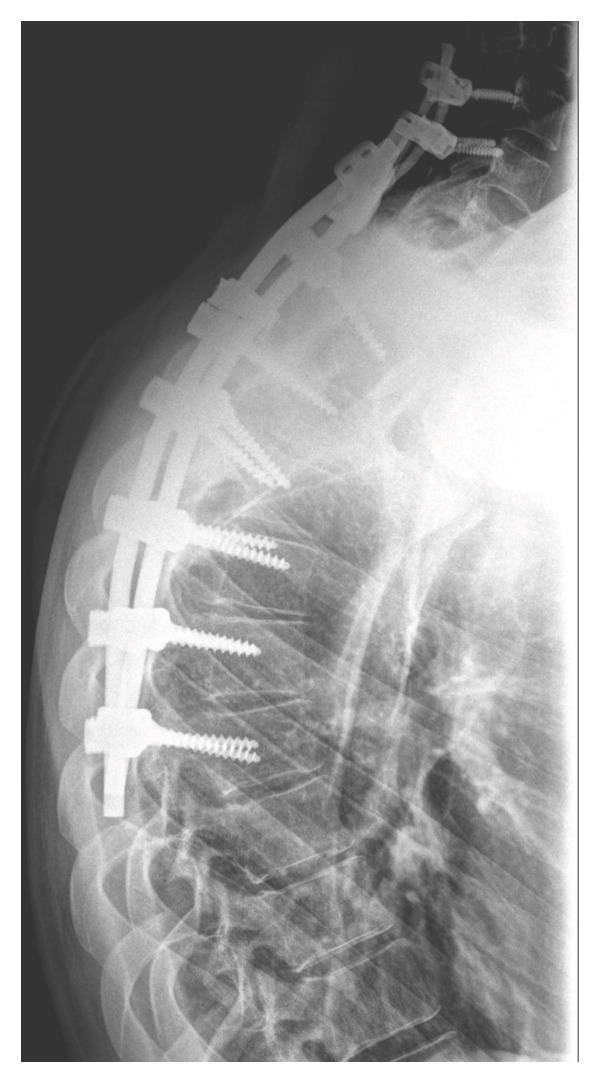
(b)

At 8 years of follow‐up, the patient noted worsening of lower limb weakness and sphincter deficiency. No other systemic symptoms were present, and the patient had no remarkable medical history.

### 2.1. Imaging Evaluation

Initial MRI of the spine was performed to differentiate between tumor recurrence and other potential diagnoses. However, significant artifacts from spinal instrumentation severely affect image quality, preventing adequate differentiation between the spinal cord and CSF, thereby precluding a definitive diagnosis.

Given these limitations, CT myelography was performed in accordance with our institutional protocol (Figure 3). The procedure was performed with the patient in the lateral decubitus position. Under local anesthesia with 2% lidocaine, a standard lumbar puncture was performed using a 22G Quincke needle. Correct positioning within the subarachnoid space was confirmed by observing continuous CSF flow, and a 10‐mL sample was obtained. Contrast administration consisted of a slow injection of 5 mL of nonionic iodine solution followed by 5‐mL saline. To facilitate contrast migration to the upper cervical segment, the patient was maintained in a supine position for 1‐2 h before undergoing standard CT imaging of the targeted spinal segments.

Figure 3CT myelography. Note enlargement of the spinal cord (arrow, (a)), decreasing anterior and posterior iodine contrast columns. (b, c) Axial slices, note the smaller iodine contrast rim between (b) and (c) (arrows).

**Figure:**
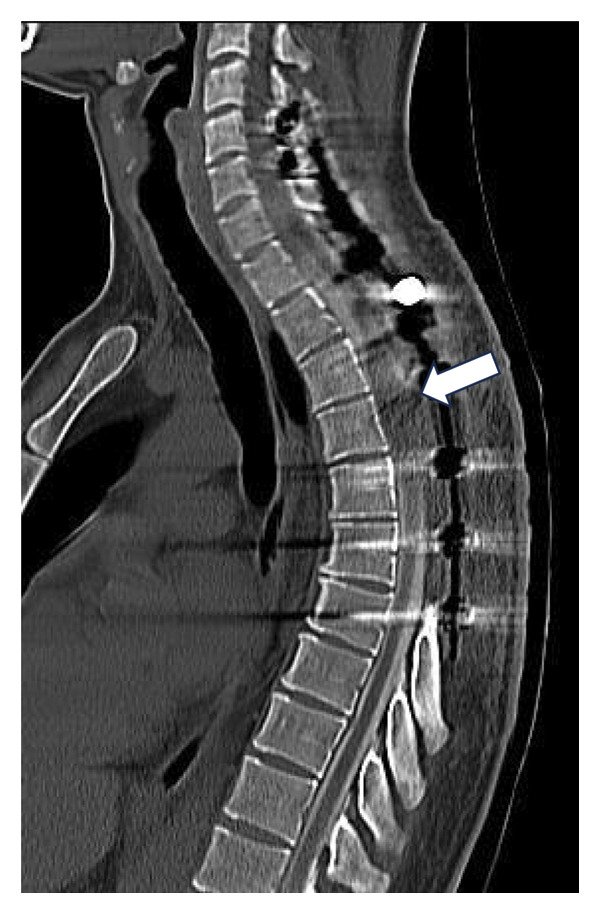
(a)

**Figure:**
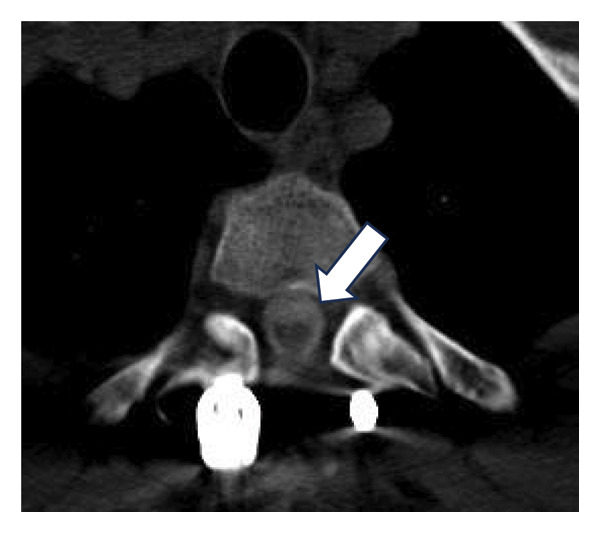
(b)

**Figure:**
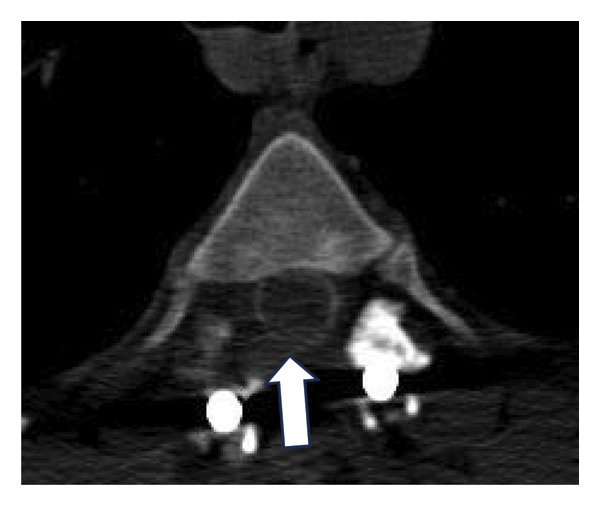
(c)

CT myelography (Figure 3) revealed abnormal enlargement of the upper thoracic spinal cord. Although this finding suggests local recurrence, alternative diagnoses such as syringomyelia should be considered.

### 2.2. Surgical Procedure

**Figure 5:**
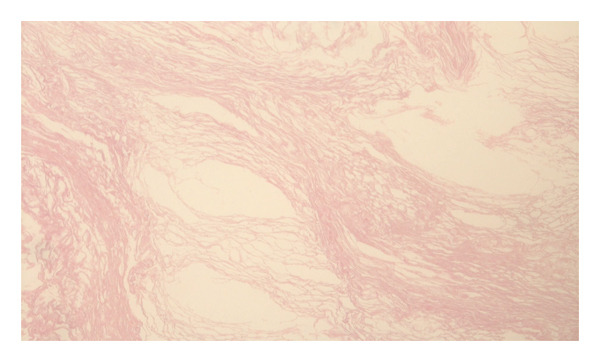
Pathological micrograph showing keratin lamellae characteristic of epidermal cysts, hematoxylin and eosin stain, 400x magnification.

The operation was performed under general anesthesia with continuous electrophysiological monitoring of somatosensory and motor‐evoked potentials. The patient was placed in the prone position, and access was achieved through a previous midline cervicothoracic incision. Meticulous dissection of the paravertebral muscles exposed the dura mater and the previous longitudinal fixation bar. A midline dural opening was performed, revealing an enlarged spinal cord at the thoracic level (Figure 4). Approaching intramedullary tumors via the posterior median sulcus provides optimal visualization while avoiding damage to the ascending adjacent tracts. The procedure revealed characteristic flaky keratin lamellae that were completely excised, along with the cyst capsule. Microscopic pathological examination (Figure 5) showed the characteristic keratin lamellae.

Figure 4Intraoperative aspect after dural sac opening. The spinal cord shows an intramedullary tumor (a) that exhibits the characteristic lamellae of keratin (b).

**Figure:**
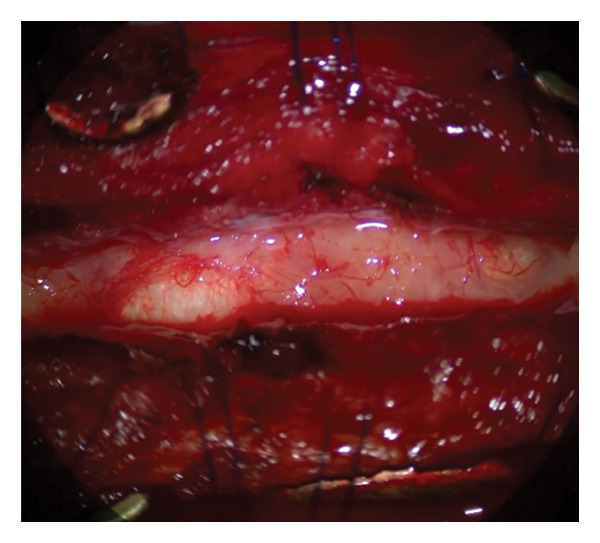
(a)

**Figure:**
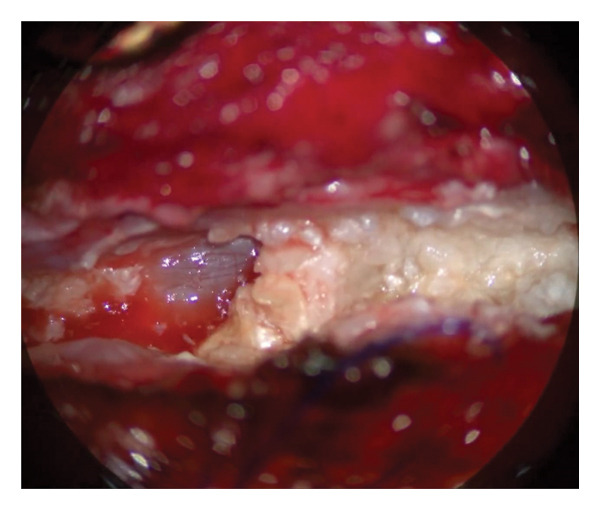
(b)

### 2.3. Follow‐Up

The surgical procedure was uneventful, and the patient was discharged without any additional neurological deficits. Subsequent follow‐up evaluations demonstrated the preservation of normal lower limb strength, although complete resolution of sphincter dysfunction was not achieved.

## 3. Discussion

Epidermoid cysts are benign, rare neoplasms, comprising less than 1% of all intraspinal neoplasms [7]. Women are slightly more compromised than males (54%) with a bimodal age distribution [6]. The disease can be either acquired, typically related to trauma or following neurosurgical procedures [8], or congenital [6], with the latter being more prevalent [6]. Similar to the present case, insidious presentation leading to late diagnosis is the rule [6, 7]. Isolated intramedullary involvement is rare, with a recent literature review reporting 39 cases [6]. Other affected locations include the intracranial and intracerebral compartments [9, 10]. Although surgical resection is the most commonly described treatment approach [6, 7], biopsy and radiotherapy are reserved for selected cases. Sirbu et al. [6] reported a spinal recurrence rate of 10.9%, which was inversely correlated with the extent of the surgical resection.

Epidermoid cysts are thought to arise from the retained ectodermal epithelium during neural tube closure between the third and fifth weeks of gestation [9], although iatrogenic implantation of skin cells following surgical procedures or diagnostic needle puncture has been reported [6, 8]. The gross pathological appearance is described as “pearly tumors,” due to their characteristic shiny “mother of pearl” aspect, presenting as well‐circumscribed, nodular surface [9]. The cyst’s content is composed of waxy or flaky material composed of debris, keratin, water, and cholesterol crystals [11] derived from progressive desquamation and keratin breakdown of stratified squamous epithelium that internally lines the cyst [9]. Malignant transformation is extremely rare, with some cases reported for lesions arising in the intracranial space [6], but only one in the spinal compartment [12]. The most common clinical presentation of intramedullary cysts is pain (both back and radicular pain), followed by motor and sensory deficits, and less commonly sphincter deficiency [6].

Spine MRI is the imaging modality of choice for diagnosis [6, 9] allowing the differential diagnosis, lesion extension evaluation, and surgical planning. Usually, MRI studies show a well‐circumscribed nodule that is isointense to slightly hyperintense on T1‐weighted sequences, with a high signal on T2‐weighted sequences [6, 9]. This classic radiological aspect might change mainly because of the variable components of the cyst’s content. Diffusion restriction inside the mass on diffusion‐weighted imaging (DWI) sequences and a thin rim of gadolinium uptake may be present [6], and differential diagnosis includes other intramedullary neoplasms, such as astrocytomas and ependymomas. The final diagnosis should be made by pathological examination of the cyst wall and its contents.

CT myelography remains valuable in the MRI era, particularly for diseases of the intradural‐extramedullary space [1]. Unlike MRI, a major limitation of CT myelography is poor soft tissue differentiation, which prevents neural tissue diseases from being readily differentiated. The best indications for CT myelography include diseases that narrow or displace the spinal cord, roots, and thecal sac [1]. Patency evaluation makes this method suitable for the management of spinal malignancies, especially leptomeningeal involvement [13]. High‐quality osseous structure visualization and surgical planning are other advantages of this method [1]. The disadvantages are mostly related to the invasiveness of the myelography procedure, which includes myelography‐induced seizures, nervous tissue injury, and iodine contrast allergy [14]. Contrast‐induced toxicity is rare, although status epilepticus, aseptic meningoencephalitis, and vasogenic brain edema have been reported [14].

## 4. Conclusion

Epidermoid cysts are rare, benign central nervous system neoplasms that predominantly affect the intracranial space [15]. An exceptionally uncommon intramedullary location clinically presents with progressive spinal cord syndrome. Local recurrence after surgical removal is infrequent [7]. Exceptionally, transformation to squamous cell carcinoma has been described in the literature [12].

Previously treated patients presenting with new or worsening neurological deficits require imaging evaluation for local recurrence. CT myelography can be used in selected cases for spinal canal evaluation, particularly in patients with extensive, artifact‐producing vertebral instrumentation. Once recurrence is confirmed, cyst capsule resection is the definitive therapeutic approach.

This case highlights the importance of long‐term surveillance in patients with intramedullary epidermoid cysts and the current utility of CT myelography in specific clinical scenarios in which conventional MRI is inadequate for diagnostic purposes.

**Nomenclature**
CT: Computed tomography
MRI: Magnetic resonance imaging
CSF: Cerebrospinal fluid
DWI: Diffusion‐weighted sequences

## Disclosure

All authors agree to be accountable for the content and conclusions of the article. No third‐party services were involved in the research or manuscript preparation who are not listed as an author and have not been acknowledged.

## Conflicts of Interest

The authors declare no conflicts of interest.

## Funding

The authors received no financial support for the research, authorship, and/or publication of this article.

## Data Availability

The data that support the findings of this study are available on request from the corresponding author. The data are not publicly available due to privacy or ethical restrictions.
